# Autophagy in Development, Cell Differentiation, and Homeodynamics: From Molecular Mechanisms to Diseases and Pathophysiology

**DOI:** 10.1155/2014/349623

**Published:** 2014-08-28

**Authors:** Ioannis P. Nezis, Maria I. Vaccaro, Rodney J. Devenish, Gábor Juhász

**Affiliations:** ^1^School of Life Sciences, University of Warwick, Coventry CV4 7AL, UK; ^2^Department of Pathophysiology, School of Pharmacy and Biochemistry, Institute of Biochemistry and Molecular Medicine, University of Buenos Aires (UBA), National Council for Scientific Research (CONICET), 1113 Buenos Aires, Argentina; ^3^Department of Biochemistry and Molecular Biology, Monash University, Clayton Campus, Clayton, VIC 3800, Australia; ^4^Department of Anatomy, Cell and Developmental Biology, Eötvös Loránd University, Budapest 1117, Hungary

The focus of this special issue is to highlight the role of autophagy in cellular homeodynamics, cell differentiation, and development with an outlook to diseases. Autophagy is an evolutionarily conserved catabolic process where cytoplasmic components are sequestered into double-membrane vesicles called autophagosomes, which then fuse with lysosomes and their content is degraded. Despite the significant progress observed over recent years in our understanding of the molecular mechanisms of autophagy, the elucidation of its role in developmental processes still remains a challenge for the scientific community. Given the role of autophagy in pathophysiology and diseases, it is essential to uncover how the mechanisms of autophagy function during developmental processes in the context of tissue and organismal physiology.

This special issue contains a collection of four original research papers, four review articles, and one methodology report, covering a broad range of topics.

A. P. Sagona et al. in their paper entitled “*Association of CHMP4B and autophagy with micronuclei: implications for cataract formation*” report that the ESCRT-III subunit CHMP4B localizes to chromosome bridges and micronuclei in lens epithelial cell cultures. These structures are subject to autophagy as evidenced by the close proximity of autophagosomes and lysosomes. Moreover, based on the observation that CHMP4B can be coimmunoprecipitated with chromatin, the authors propose that CHMP4B contributes to selective autophagy, here leading to the degradation of micronuclei and other extranuclear chromatin. As a CHMP4B mutation associated with an autosomal dominant form of cataract abolishes the ability of CHMP4B to localize to micronuclei, the autophagic degradation of DNA is implicated in the protection of lens cells from cataract development.

K. Zielniok et al. in their paper entitled “*Functional interactions between 17*β*-estradiol and progesterone regulate autophagy during acini formation by bovine mammary epithelial cells in 3D cultures*” use a 3D culture model of developing mammary epithelial cell acini to elucidate the mechanisms of autophagy regulation by 17*β*-estradiol and progesterone. They investigate the genomic effect of both sex steroids on the expression of chosen autophagy-related genes and proteins (ATGs). They show that both hormones induce ATG3, ATG5, BECN1, LC3B, and their protein products during the formation of alveoli-like structures by bovine BME-UV1 mammary epithelial cells. Moreover, the treatment with both hormones slightly increased the level of phosphorylated AMPK but diminished phosphorylated Akt and mTOR on day 9 of 3D culture. These results also suggest that the synergistic actions of the two steroid hormones studied accelerate the development of bovine mammary acini, which may be connected in part with the regulation of the molecular machinery involved in autophagy.

K. Hegedűs et al. in their paper entitled “*The putative HORMA domain protein Atg101 dimerizes and is required for starvation-induced and selective autophagy in Drosophila*” study the Atg1-family kinase complex in* Drosophila* and identify Atg101 as a member of this complex. They show that loss of* Drosophila* Atg101 impairs both starvation-induced and basal autophagy. They also show that Atg101 dimerizes and is predicted to fold into a HORMA domain. In addition Atg101 interacts with Atg1, Atg13, and Ref(2)P. These results suggest an important role of* Drosophila *Atg101 in autophagy and highlight that the Atg1 kinase complex is conserved among the metazoans.

P. Lőrincz et al. in their paper entitled “*Atg6/UVRAG/Vps34-containing lipid kinase complex is required for receptor downregulation through endolysosomal degradation and epithelial polarity during Drosophila wing development*” analyse the role of class III phosphatidyl-inositol 3-kinase (PI3K) complexes during wing development in* Drosophila*. The core complex consists of the lipid kinase Vps34 and its regulatory subunit Vps15, plus Atg6/Beclin1. Distinct PI3K complexes are specified by the two mutually exclusive subunits Atg14 and UVRAG. It is shown that both Atg6 and Atg14, but not UVRAG, are required for autophagy in the wing, whereas both Atg6 and UVRAG, but not Atg14, are involved in the endolysosomal degradation of receptors, such as Notch, and also in the establishment of proper epithelial polarity. There is some controversy regarding the role of UVRAG in autophagy in the published literature. This study supports a series of papers showing the existence of an autophagy-specific Atg14 complex, while the UVRAG-containing complex is found to be necessary for endolysosomal degradation and cell polarity.

The review by N. C. Mulakkal et al. entitled “*Autophagy in Drosophila: from historical studies to current knowledge*” comprehensively summarises the role and regulation of autophagy in the fruit fly,* Drosophila melanogaster*, an established model system for in vivo analysis of autophagy in the context of a developing organism. Autophagy genes and their regulators are conserved in* Drosophila*, and autophagy is induced in response to nutrient starvation and hormones during development. Application of sophisticated genetic tools allows investigation of autophagy in* Drosophila* models of disease. The power of the* Drosophila* model means it has made important contributions to the identification of novel developmental and physiological roles of autophagy.

The review by D. Romanelli et al. entitled “*A molecular view of autophagy in Lepidoptera*” focuses on Lepidopteran insects including the silkmoth* Bombyx mori*, which are classical subjects of autophagy research. Enormous induction of autophagy is seen in the polyploid larval tissues of these animals in response to starvation or during metamorphosis, similar to* Drosophila.* A key advantage of studying Lepidopteran models is their direct economic impact. For example, insects belonging to this order can produce silk or decrease crop yields as pests. Recent advances in developing tools for molecular studies hold the promise that the analysis of autophagy and programmed cell death in Lepidopteran larvae (caterpillars) may shed light onto the role and regulation of these processes in a developmental context.

The review by J. M. I. Barth and K. Köhler entitled “*How to take autophagy and endocytosis up a Notch*” summarizes the interplay and published links between two catabolic pathways: endocytosis and autophagy, both of which culminate in lysosomal degradation. The established role of endocytosis in regulating Notch receptor activity and the availability of its ligands Delta, Serrate, and Lag-2 is also discussed, together with emerging data on autophagy as a modulator of Notch signaling. Vice versa, loss of Notch leads to the activation of autophagy in certain contexts. Considered together these data indicate a complex network of interactions between autophagy, endocytosis, and Notch signaling, which is only beginning to be understood.

The review by M. Lippai and P. Lőw entitled “*The role of the selective adaptor p62 and ubiquitin-like proteins in autophagy*” provides a brief overview of autophagy and the ubiquitin-proteasome system and how these degradation systems coordinate their functions. They highlight the presence of ubiquitin and ubiquitin-like proteins in both systems and discuss the basic mechanisms of their function. Moreover, the authors underscore the selectivity of degradation in both systems and focus extensively on selective autophagy and its associated adaptor proteins including p62/SQSTM1, NBR1, NDP52, and Optineurin. The involvement of ubiquitin and ubiquitin-like proteins of Atg8 family in selective autophagy is also discussed.

A. L. Kovács in his paper entitled “*A simple method to estimate the number of autophagic elements by electron microscopic morphometry in real cellular dimensions*” describes a morphometric method (S_sp_ method) for calculation of surface values and estimation of average diameter and number of autophagic elements in real cellular dimensions using data from electron micrographs. The method is based on morphometric determination of relative surface (surface density) and volume (volume density). Since electron microscopy is still indispensable for autophagy research, the S_sp_ method will be very useful for providing quantitative analysis of electron microscopy data.

In conclusion, the papers presented in this special issue underscore a prominent role of autophagy during cell differentiation and development and highlight its emerging association with diseases. A deeper understanding of the mechanisms of autophagy in the context of developmental processes thus emerges as a critical factor for the development of novel therapeutic approaches.

*Ioannis P. Nezis* *Maria I. Vaccaro* *Rodney J. Devenish* *Gábor Juhász*

